# Racial Discrimination Moderates the Association Between Alcohol Consumption and Alcohol-Related Consequences Among One First Nation Reserve Community’s Youth

**DOI:** 10.1007/s40615-025-02637-w

**Published:** 2026-07-29

**Authors:** Tessa Nalven, Melissa R. Schick, Nichea S. Spillane

**Affiliations:** 1 PATHS Lab, Department of Psychology, University of Rhode Island, 110 Chafee Hall, 142 Flagg Rd, Kingston, RI 02881 USA; 2 School of Public Health, Brown University, Providence, RI USA; 3 School of Medicine, Yale University, New Haven, CT USA; 4 Department of Psychology, Arizona State University, Tempe, AZ USA

**Keywords:** Indigenous, First Nation, Adolescents, Racial discrimination, Alcohol consumption, Alcohol-related consequences

## Abstract

North American Indigenous (NAI) adolescents experience disproportionately high rates of alcohol-related consequences and racial discrimination but report similar rates of alcohol consumption as other racial/ethnic groups. Racial discrimination serves a deleterious role on adolescent development and well-being. To test if NAI adolescents’ disproportionate rates of alcohol-related consequences are related to the negative effects of racial discrimination, the present study examined whether racial discrimination moderates the association between alcohol consumption and alcohol-related consequences among NAI adolescents. Participants were First Nations adolescents from reserve communities in Eastern Canada (*n* = 60, *M*_*age*_ = 15.9, 53.3% female) who reported lifetime alcohol use. The interaction of alcohol consumption by racial discrimination was statistically significantly and positively associated with alcohol-related consequences (*b* = 1.28, *SE* = 0.56, *p* = .025). Simple slopes analyses revealed that alcohol consumption was significantly positively related to alcohol-related consequences for those reporting high levels of racial discrimination (1 *SD* above the mean; *b* = 1.19, *SE* = 0.27, *p* < .001), but not for those reporting relatively low levels of racial discrimination (1 *SD* below the mean). Thus, racial discrimination may contribute to the disproportionate rate of consequences related to alcohol consumption experienced by NAI adolescents. These findings suggest the need for systems-level interventions to address racial discrimination and prejudice, as well as individual-focused interventions to increase factors previously found to protect against the negative effects of racial discrimination (e.g., racial-ethnic socialization, self-compassion).

## Introduction

North American Indigenous (NAI^1^) adolescents report rates of alcohol consumption that are high but may be comparable to other racial groups; however, findings are mixed, likely due to the vast heterogeneity amongst different NAI cultural and regional groups. For instance, a large epidemiologic study found that NAI adolescents report disproportionately high rates of lifetime alcohol consumption compared to the general population (i.e., Monitoring the Future; [1]). Yet, other studies have found NAI individuals abstain from alcohol consumption at higher rates [2] or that NAI adolescents report equal levels of lifetime alcohol consumption [SAMHSA; [3]] compared to other racial/ethnic groups. Of great public health relevance, despite the overall variability in reporting of alcohol consumption rates, research has made evident that NAI youth experience disproportionately high rates of alcohol-related consequences compared to other racial/ethnic groups [4–8]. These consequences cut across many areas, including social, legal, and occupational problems. For example, NAI youth are more likely to get in legal, school, and work trouble, or have relationship problems such as fights with parents or friends due to their alcohol consumption and are three times more likely to get a traffic ticket or be in a car accident because of alcohol consumption compared to White youth [9]. Notably, these consequences were found to be even more pronounced among NAI youth living on a reservation [9]. These disproportionate consequences related to alcohol consumption experienced by NAI adolescents are of great importance, as they cut across many important aspects of youths’ lives and therefore likely have a strong influence on adolescents’ development and growth throughout adulthood. Given that rates of alcohol consumption in NAI youth may not largely differ from other racial/ethnic groups, there are likely some factors influencing the link between alcohol consumption and consequences and leading to the disproportionately high rates of alcohol-related consequences that are experienced by NAI youth.

A well-known theory to explain these health inequities among NAI youth, focuses on the historical context of NAI people’s relationships with alcohol and colonialism. Colonization stripped NAI people of their language, culture, and lands, which has had negative intergenerational effects (i.e., historical trauma; [10]) and has effected the way NAI people consume alcohol. Colonizers intentionally distributed alcohol to NAI communities as a means of causing harm and in order to take over resources [11]. Combined with the forced cultural destruction, the historical and ongoing oppression faced by NAI people has left communities with a lack of access to traditional ways of healing and coping [12]. Racial discrimination, defined as unfair treatment by individuals and/or social institutions because of one’s race [13], is one such contributing factor that relates to the disproportionate alcohol-related consequences experienced by NAI youth. Notably, racial discrimination is highly prevalent among NAI individuals [14]. One large, epidemiological study in Canada found that as many as 73% of NAI individuals believe that they are treated unfairly because of their race or culture, and Canadians report that they believe NAI people experience the most frequent discrimination in society today [15]. Racial discrimination has been found to play an important and detrimental role in adolescent development among non-White groups broadly [for a meta-analytic review, see [16]] and among NAI youth in particular [17, 18]. For NAI youth, it is theorized that historical loss and ongoing racial discrimination are among the main explanatory variables for health disparities, and alcohol-related disparities, experienced by NAI communities [19, 20].

Empirical studies have supported such theories in that racial discrimination has been separately linked to either alcohol consumption or alcohol-related consequences. Racial discrimination has been associated with problematic alcohol consumption in nationally representative samples including NAI individuals [21]. Two longitudinal analyses of NAI adolescents found that racial discrimination predicted increased risk of heavy alcohol use and the later development of an alcohol use disorder [22, 23]. In a sample of NAI adolescents, Dickerson and colleagues [17] found that racial discrimination was related to alcohol-related consequences after controlling for the effects of gender and age; however, their study did not control for differences in alcohol consumption. While not among NAI youth, research among African American college freshmen suggests that racism-related stress was significantly associated with alcohol-related consequences, even after controlling for alcohol consumption and other important covariates [24]. While there are notable differences in NAI and African American communities and their experiences with colonialism and oppression, it is noteworthy that stress from such racism-related experiences is related to consequences with alcohol use, even when controlling for alcohol use rates. Given that previous research has found drinking to cope with negative affective states, such as those that may arise due to racism-related stress, is associated with increased risk of experiencing negative alcohol-related consequences [25], racial discrimination may influence the likelihood of experiencing negative alcohol-related consequences due to the heightened distress it may cause [26]. Alternatively, it may be that racial discrimination directly increases the risk that youth will experience negative alcohol-related consequences because of racist systematic and structural inequalities, rather than indirectly influencing the likelihood of experiencing consequences through more harmful patterns of alcohol consumption. For instance, NAI youth may be more likely to experience legal consequences from alcohol consumption simply because of racial inequities and racism endemic to the criminal justice system. For example, NAI adolescents are nearly four times as likely as White adolescents to be arrested and committed to juvenile justice facilities despite the fact that they are not at increased likelihood of committing crimes [27]. While previous studies have linked racial discrimination to alcohol consumption and alcohol-related consequences separately in NAI individuals, no studies to date have tested how racial discrimination might influence the relation between alcohol consumption and consequences. Therefore, there is a critical need for research that examines how racial discrimination might influence the relation between alcohol consumption and consequences.

The present study aims to extend previous research by specifically examining the moderating role of racial discrimination on the relationship between alcohol consumption and alcohol-related consequences among one community’s reserve-dwelling First Nation adolescents who report having had at least one full drink of alcohol in their lifetime. We hypothesized that racial discrimination would moderate the relationship between alcohol consumption and alcohol-related consequences such that the relationship between alcohol consumption and alcohol-related consequences would be stronger among NAI youth reporting high (but not low) levels of racial discrimination.

## Methods

### Participants and Procedures

The present study consists of secondary analysis of data collected for a community-based participatory research (CBPR) study that aimed to examine risk and protective factors associated with substance use among First Nation adolescents [28]. As a CBPR study, a community advisory board including community healthcare workers and elders, as well as tribal council, were involved in the parent study design, data collection and management, and dissemination. Participants (i.e., First Nation adolescents between the ages of 10 and 18 living in a reserve community) were recruited through advertisements and announcements in reserve communities and by snowball sampling (i.e., word of mouth). Research procedures were approved by institutional review board and Tribal Chief and Council prior to beginning data collection. After parent permission was obtained, adolescents who participated in the study also provided assent. Of note, all prospective participants who approached study staff and expressed an interest in participating were found to be eligible to participate, were given parent permission, and provided assent to participate. In 2017, pencil-and-paper surveys were administered and took approximately 45 min to complete; participants were compensated $25. A total of 106 adolescents who identified as First Nation and who reside in a rural First Nation reserve community in Southern and Eastern Canada were included in the parent study; the present study comprises a subset of that sample who endorsed lifetime alcohol consumption (i.e., reported having consumed at least one full alcoholic drink in their lifetime; *n* = 60). Those who endorsed never having had a full alcoholic drink were excluded from the study analyses (*n* = 46).

### Measures

#### Demographics

Participants self-reported their age in years and sex (coded such that 0 = *male* and 1 = *female*). Parents of adolescents completed measures including their total annual household income and alcohol use as assessed by the 10-item validated measure of alcohol use and related problems, Alcohol Use Disorders Identification Test (AUDIT) [29].

#### Alcohol Consumption

Lifetime Alcohol Use was assessed with one question asking if participants had ever had one full alcoholic drink. Responses were coded so that 0 = *no* and 1 = *yes*. The present sample was selected such that only participants who responded “yes” were included for further analyses.

Alcohol Consumption was assessed using the four-item Adolescent Drinking Questionnaire [ADQ; [30]], a self-report measure examining the frequency of alcohol consumption, high-volume drinking (i.e., more than 4/5 drinks for females/males per occasion), drinking until intoxicated, and average number of alcoholic drinks consumed per drinking day over the past three months. Participants rated each item on an eight-point scale: frequency items ranged from 0 = *never* to 7 = *everyday* and quantity items ranged from 0 = *1 standard drink* to 7 = *more than 10 standard drinks*. Item scores were summed to create a total score with higher scores reflecting greater alcohol consumption. Cronbach’s α in the current sample was good, α = 0.87.

#### Racial Discrimination

Racial discrimination was assessed using 10 self-report items measuring a range of potential types and sources of racial discrimination (e.g., being treated unfairly because of their race) for American Indian adolescents, including discrimination by authority figures or in a store [26, 31]. The original measure was adapted by replacing “American Indian” with “First Nation” to match this sample’s racial identification. Participants rated each item on a three-point scale from 0 = *never* to 2 = *many times.* Items scores were averaged to create a combined score with higher scores reflecting greater racial discrimination. This scale has been found to have good psychometric properties in previous research with NAI adolescents [32, 33]. Cronbach’s α in the present study was good, *α* = 0.86.

#### Alcohol-Related Consequences

Alcohol-related consequences were assessed using the 23-item Rutgers Alcohol Problem Index [RAPI; [34]], a self-report measure for adolescents that assesses the frequency of experiencing negative consequences (e.g., social, emotional, physical, etc.) in the past year because of alcohol consumption (e.g., “neglected your responsibilities” or “passed out or fainted suddenly”). Participants rated each item on a four-point frequency scale ranging from 0 = *never* to 3 = *more than 5 times*. Item scores were summed to create a total score with higher scores reflecting more frequent experiences of consequences due to alcohol consumption. The RAPI has demonstrated good psychometric properties in previous research with First Nation individuals [35]. Cronbach’s *α* in the present study was excellent, *α* = 0.93.

### Analytic Strategy

All analyses for the present study were conducted in IBM SPSS Statistics v.26. First, descriptive statistics, frequencies, and bivariate correlations (i.e., Pearson product-moment correlations among continuous variables and point-biserial correlations among dichotomous and continuous variables) were examined to assess for assumptions of normality, homoscedasticity, and multicollinearity [36, 37]. Then, to test whether racial discrimination moderates the association between alcohol consumption and alcohol-related consequences, a moderation analysis was conducted using model one of the SPSS PROCESS macro [38]. Age and sex were included as covariates in the model given previous literature suggesting that likelihood of substance use increases over the course of adolescence [39] and differs based on sex [40]. Predictor variables were mean centered prior to the construction of the interaction term to aid in interpretation of parameter estimates and to lessen the correlation between the interaction term and its component variables [38]. The PROCESS procedures use bootstrapping and ordinary least squares regression to estimate the model coefficients for all analyses. These methods confer more statistical power than do standard approaches to statistical inference and do not rely on distributional assumptions [38]. Bootstrapping was done with 5,000 random samples generated from the observed covariance matrix to estimate bias-corrected 95% confidence intervals (CIs) and significance values. Using the methods described by Aiken, West, and Reno [41], the significant interaction was followed up with simple slopes analyses; differences in regression slopes for alcohol consumption were plotted among participants with relatively high (one *SD* above the mean) and low (one *SD* below the mean) levels of racial discrimination. Of note, because we are examining alcohol-related consequences, we limited our sample to only those who reported having had a full drink of alcohol in their lifetime to reduce zero-inflation from those who report no consequences from alcohol consumption because they have not consumed any alcohol.

## Results

### Preliminary Analyses

Participants for the present study ranged in age from 12–18 years (*M* = 15.9, *SD* = 1.7) and were 53.3% female (*n* = 32) and 46.7% male (*n* = 28). Parental data (*n* = 133) suggests that the average total yearly household income was between $20,000–39,000 and the modal income was $0–9,999. 66.9% (*n* = 89) of parents reported drinking alcohol monthly or less and 47.6% (*n* = 50) reported drinking 5 or more drinks per drinking day. Scores for primary variables of interest were approximately normally distributed based on established guidelines that absolute values of skewness > 2 and kurtosis > 4 indicate non-normality [42, 43]. See Table 1 for all descriptive statistics and bivariate correlations among alcohol consumption, racial discrimination, alcohol-related consequences, and covariates. Of note, descriptive statistics revealed that 55 (93.2%) NAI youth reported having experienced any racial discrimination, 19 (34.5%) reported binge alcohol use (4/5 drinks per occasion for female/male youth) at least monthly or more frequently in the past three months, and 42 (72.4%) reported experiencing at least one alcohol-related consequence in the past year. Pearson product-moment correlations revealed significant positive correlations between alcohol consumption and alcohol-related consequences (*r* = 0.56, *p* < 0.001) and between racial discrimination and alcohol-related consequences (*r* = 0.29, *p* = 0.029). However, alcohol consumption was not significantly associated with racial discrimination (*r* = 0.20, *p* = 0.134).

**Table 1:** Descriptive statistics and bivariate correlations among variables of interest

	1	2	3	4
1. Alcohol consumption	-			
2. Racial discrimination	.199	-		
3. Alcohol-related consequences	.559***	.290*	-	
4. Age	.363**	-.135	.183	-
5. Female sex^1^	-.061	.179	-.037	-.033
*M*	6.32	0.63	9.53	15.86
(SD)	(6.34)	(0.43)	(12.42)	(1.71)
Range	0–28	0–2	0–69	12–18

*p <.05, ** *p *<.01, ***p <.001

^1^Reference group is to male sex

### Moderation Analysis

**Fig. 1:**
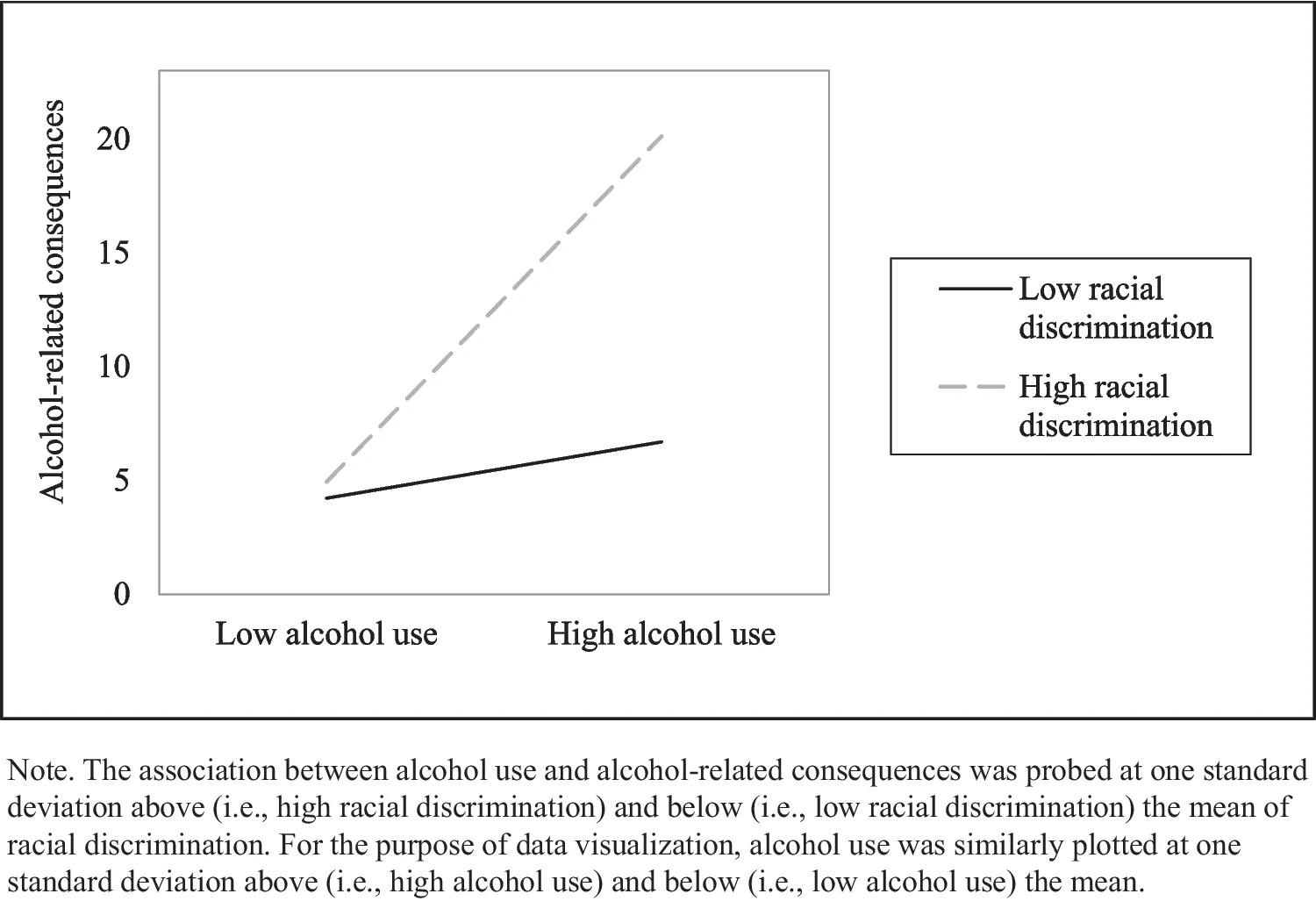
Alcohol consumption by racial discrimination interaction for alcohol-related consequences

Moderation analysis examined the main and interactive effects of alcohol consumption and racial discrimination on alcohol-related consequences, as summarized in Table 2. The overall model for alcohol-related consequences was statistically significant, *F*(5, 49) = 7.82, *p* < 0.001, *R*^*2*^ = 0.44. Statistically significant and positive main effects were detected for alcohol consumption (*b* = 0.69, *SE* = 0.27, *p* = 0.013) and racial discrimination (*b* = 9.12, *SE* = 4.05, *p* = 0.029). Moreover, the interaction of alcohol consumption by racial discrimination was statistically significantly positively associated with alcohol-related consequences (*b* = 1.28, *SE* = 0.56, *p* = 0.025). As illustrated in Fig. 1, analysis of simple slopes revealed that alcohol consumption was statistically significantly positively related to alcohol-related consequences for those reporting relatively higher levels of racial discrimination (1 *SD* above the mean; *b* = 1.19, *SE* = 0.27, *p* < 0.001) but was not statistically significantly related to alcohol-related consequences for those reporting relatively lower levels of racial discrimination (1 *SD* below the mean; *b* = 0.19, *SE* = 0.41, *p* = 0.637).

**Table 2:** Interaction of alcohol consumption by racial discrimination for alcohol-related consequences

	*b*	*SE*	*t*	*p*	95% CI
Constant	2.92	14.93	0.20	.846	[−27.08, 32.91]
**Alcohol Consumption**	**0.69 **	**0.27 **	**2.58**	**.013**	**[0.15, 1.23] **
**Racial discrimination**	**9.12**	**4.05**	**2.25**	**.029**	**[0.97, 17.26] **
Female Sexa	−3.36	2.88	−1.17	.249	[−9.14, 2.42]
Age	0.50	0.94	0.53	.601	[−1.40, 2.39]
**Alcohol consumption X racial discrimination**	**1.28**	**0.56**	**2.30**	**.025**	**[0.16, 2.39]**

^a^Male is reference group

Bolded typeface indicates significance at the *p* <.05 level

### Sensitivity Analyses

Given the relatively small sample size, we conducted post hoc sensitivity analyses using G*Power to examine whether analyses were adequately powered to detect effects [44]. Analyses revealed that we were adequately powered to detect effects as low as Cohen’s *d* = 0.47, which corresponds to *r* = 0.23 and *R*^*2*^ = 0.05. Effects observed in the present study were above this threshold.

## Discussion

The present study was the first to explore the moderating role of racial discrimination on the relationship between alcohol consumption and alcohol-related consequences in First Nation adolescents living in one reserve community. The findings are noteworthy given the disproportionate health disparities related to alcohol consumption experienced by NAI adolescents [4–8], and results offer important targets for intervention and public health implications.

The results revealed that racial discrimination and alcohol consumption were both related to alcohol-related consequences in bivariate and multivariate analyses. These findings are not surprising and are consistent with previous literature finding that those who consume more alcohol or engage in riskier drinking will experience more related consequences [45, 46] and that racial discrimination is associated with alcohol-related consequences among NAI youth [17]. Our results are also similar to findings from Grekin (2012) who found that, among African American college students, experiences of racism were related to alcohol-related consequences, even after controlling for the effects of alcohol consumption. The present study’s results may help to understand why, even though rates of alcohol consumption do not differ overall across White and NAI adolescents, NAI youth experience greater consequences related to alcohol consumption. While explanatory factors between racial discrimination and consequences have not been tested in this study, it may be that NAI youth experience distress following experiences of racial discrimination [26], which leads to harmful patterns of alcohol consumption in order to cope, increasing subsequent risk for experiencing negative consequences [25]. Thus, it may be that racial discrimination contributes to disparities in alcohol-related consequences observed across racial groups, given our finding that racial discrimination was significantly related to alcohol-related consequences, even after accounting for individual variation in alcohol consumption.

Consistent with our hypothesis, results suggest that NAI youth who consume alcohol report high levels of alcohol-related consequences at relatively higher levels of racial discrimination. While we are the first to do this analysis and cannot compare to other research, this finding may further support the notion that racial discrimination is a contributing factor toward understanding why NAI youth report higher rates of alcohol-related consequences than their White counterparts, despite similar rates of alcohol consumption. This finding can perhaps be explained by the use of externalizing coping behaviors, such as using alcohol, in response to the distress caused by racial discrimination [26], which is notable given that using alcohol to cope is directly associated with more negative alcohol-related consequences and this relationship is not mediated by quantity and frequency of alcohol consumption (Merrill et al., 2014). Results suggest that addressing racial discrimination would be important in reducing the health disparities experienced by NAI youth. Specifically, racial discrimination and prejudice should be addressed at a systemic level to reduce its occurrence. While there are notable barriers to implementation, anti-racist trainings could be taught across all people of authority and public influencers such as educators, police, politicians, and researchers. For example, there are already interventions that target the implicit bias [e.g., the Racial Harmony Workshop; [47]] that is thought to underlie racism [48, 49], and the ongoing discriminatory acts against individuals from minoritized groups (including NAI individuals) suggest that further systemic change is needed. Moreover, community interventions that inform NAI communities about the potential negative effects of racial discrimination and that offer a supportive environment in the context of racial discrimination while suggesting alternative healthy ways of coping may be beneficial. Finally, community-level interventions to reduce access and availability of alcohol to youth could potentially serve as an important area for intervention. For example, research has shown that both community [50] and parental monitoring of youth alcohol use and parental communication about the harms related to substance use have been shown to be protective against use [51], suggesting that interventions to educate families and communities on sending messages to youth to disapprove of alcohol use and monitor access could be effective in reducing youths’ use.

Our findings may also offer important individual level implications, as certain factors are known to protect against the negative impact of racial discrimination. Parental racial-ethnic socialization of their children should be encouraged, as it can be helpful in promoting resilience to racial discrimination, particularly when cultural socialization (i.e., promoting positive views about a child’s culture and racial/ethnic identity) and preparation for bias (i.e., informing children about the existence and effects of discrimination and racism) are taught alongside promotive emotional and behavioral coping skills [52, 53]. Previous research has found that racial/ethnic identity mitigates the effects of racial discrimination, which supports that racial/ethnic identity development is important in protecting against the negative effects from racial discrimination for minoritized youth [54]. Specifically, among NAI populations, theoretical and empirical research has suggested that culture is healing (Brown et al., 2016). Cultural healing may be a particularly important area for intervention among NAI communities given the context in which these communities exist, having experienced historical trauma related to colonization that resulted in the destruction of culture due to forced removal from Tribal lands and placement of NAI children in boarding schools (Ross et al., 2015; Whitbeck et al., 2004; Wiechelt et al., 2012). Indeed, research suggests that holding strong values for cultural activities is protective [55]. Finally, interventions that promote positive self-image, such as self-esteem, self-worth, and self-compassion may promote resilience from racial discrimination; indeed, one research study found that self-compassion protects against the negative effects of racial discrimination on alcohol-related consequences in a sample of NAI youth [56].

### Limitations and Future Directions

While results of the present study contribute important knowledge, they should be considered within the context of their limitations. First, given that the data are cross-sectional and correlational in nature, causal and temporal ordering of these associations cannot be determined. Future research should examine these associations in prospective, longitudinal designs to determine the temporal relations of study variables. Second, given the vast heterogeneity of NAI groups, and that these data were collected from one band of reserve-dwelling First Nation adolescents, findings may not generalize to other Indigenous groups or to adolescents living off-reserve or in urban areas because they may differ in alcohol consumption rates, healthcare access, cultural practices, and other important areas. However, it is noteworthy that, while our sample was relatively small, based on estimates from 2016 Canadian census data [57], the present sample represents approximately one-third of the population of interest (i.e., all adolescents within this age range from this Tribe) who report having consumed alcohol. Nonetheless, future studies should seek to replicate these findings, particularly among youth in other reserve communities or with other Indigenous populations to see if findings remain true. In addition, because the present analyses were secondary analyses of existing data, we were not able to examine the moderating effects of other factors that may explain the relationship between alcohol use and consequences and that are related to the negative effects of oppression, such as historical trauma or internalized racism. Future research should consider examining other factors as they relate to consequences from alcohol use. Finally, other well-known correlates of alcohol consumption and alcohol-related consequences and outcomes related to experiencing racial discrimination were not included in the present study because they were not collected within the same dataset (e.g., socioeconomic status, mental health, parental education, and parental alcohol use). Future work should aim to incorporate other such factors to more fully elucidate these associations and provide a fuller picture of results.

## Conclusion

The present study highlights the importance of systemic level interventions that promote anti-racism and should be implemented to increase societal level understanding of bias and subsequent racial discrimination, given the potential harms it may relate to in NAI youth and their alcohol-related consequences. Researchers should, subsequently, seek to create and test family and community interventions that seek to provide coping strategies for NAI youth that promote resilience in the face of racial discrimination. Specifically, they should incorporate parental ethnic-racial socialization that focuses on promoting positive views of NAI culture, as well as preparing NAI youth for the realities of racial discrimination and ways to cope effectively with racial discrimination, such as by promoting value in cultural activities and positive self-images. Such interventions may lead to a reduction in the disproportionate alcohol-related consequences that are linked with disparities seen in the underserved NAI communities.

## Data Availability

The data that support the findings of this study are available from the corresponding author upon reasonable request.

## Footnotes

1 NAI = North American Indigenous; referring to individuals from the Indigenous groups of North America, including American Indians and Alaska Natives in the United States and First Nations, Métis, and Inuit in Canada, among others.
